# Hit-and-Run Epigenetic Editing for Vectors of Snail-Borne Parasitic Diseases

**DOI:** 10.3389/fcell.2022.794650

**Published:** 2022-02-28

**Authors:** Nelia Luviano, David Duval, Wannaporn Ittiprasert, Jean-Francois Allienne, Geneviève Tavernier, Cristian Chaparro, Celine Cosseau, Christoph Grunau

**Affiliations:** ^1^ IHPE, Univ Perpignan Via Domitia, CNRS, Ifremer, Univ Montpellier, Perpignan, France; ^2^ Department of Microbiology, Immunology and Tropical Medicine, School of Medicine and Health Sciences, George Washington University, Washington, DC, United States; ^3^ Research Center for Neglected Diseases of Poverty, School of Medicine and Health Sciences, George Washington University, Washington, DC, United States; ^4^ Transgenesis Core Facility of UMS006/Inserm/Paul Sabatier University/National Medical Veterinary School, Toulouse, France; ^5^ Inserm UMR 1048, Paul Sabatier University, Toulouse, France

**Keywords:** DNA methylation, methylome editing, vector snail, Schistosoma, Biomphalaria

## Abstract

Snail-borne parasitic diseases represent an important challenge to human and animal health. Control strategies that target the intermediate snail host has proved very effective. Epigenetic mechanisms are involved in developmental processes and therefore play a fundamental role in developmental variation. DNA methylation is an important epigenetic information carrier in eukaryotes that plays a major role in the control of chromatin structure. Epigenome editing tools have been instrumental to demonstrate functional importance of this mark for gene expression in vertebrates. In invertebrates, such tools are missing, and the role of DNA methylation remains unknown. Here we demonstrate that methylome engineering can be used to modify *in vivo* the CpG methylation level of a target gene in the freshwater snail *Biomphalaria glabrata,* intermediate host of the human parasite *Schistosoma mansoni*. We used a dCas9-SunTag-DNMT3A complex and synthetic sgRNA to transfect *B. glabrata* embryos and observed an increase of CpG methylation at the target site in 50% of the hatching snails.

## Introduction

The capacity of parasites to infect their host and the capacity of the latter to defend themselves against such intrusion is dependent on environmental factors and based on heritable components, with genetic polymorphisms as one of the major contributors to the heritable variations in virulence and resistance. However, it is now evident that there is a nonnegligible proportion of non-genetic heritable variation. Among them, epigenetic factors being probably the most important. We define here epigenetic variation as any change in chromatin structure that is at least mitotically heritable. Almost 10 years ago, Gomez-Diaz and others underlined the importance of epigenetics in host-pathogen interactions and designed the vision of a roadmap to elucidate their importance (Gómez-Díaz et al., 2012). Their concluding remarks were that “…cutting-edge epigenomic techniques combined with experimental (whole organism), functional, and theoretical (modeling) approaches will provide fascinating insights into the interrelations between genetic, epigenetic, and phenotypic variation in the complex world of host–pathogen relationships.”

Schistosomiasis is a parasitic disease caused by helminths of the genus *Schistosoma* that affects about 230 million people worldwide. Intestinal schistosomiasis originated from the African continent but is now endemic also in America and Europe and considered an emerging infectious disease there. Schistosomes have a complex life cycle with two consecutive obligatory hosts. Human dwelling larvae (cercariae) are produced by asexual multiplication in a freshwater snail intermediate host and infect the human and/or rodent definitive host when in contact with freshwater (Pearce and MacDonald, 2002; Colley et al., 2014). The parasite *S. mansoni* can be maintained in the laboratory using mice as the definitive host. Parasite eggs are then recovered from infected mouse livers and when exposed to light hatch the miracidia larvae (Galinier et al., 2017) that are used to experimentally infect juvenile snails or that are transformed into sporocysts *in vitro* as previously described (Roger et al., 2008). If snails are infected with miracidia, 2 weeks later the prevalence and intensity of infection can be assessed. Maximum compatibility is reached in sympatric host/parasite combinations (Theron et al., 2014).

Schistosomiasis leads often to anemia, growth stunting, exacerbation of co-infections, and impairment of cognitive development and work capacity (King and Dangerfield-Cha, 2008). No vaccine exists to prevent this pathology. The means of control are based primarily on mass treatment campaigns in human communities through the use of Praziquantel, the only molecule partially effective against schistosomes (Gryseels et al., 2006; Herricks et al., 2017). However, this molecule does not protect against inevitable re-infections in endemic areas. Thus, 78 million patients received treatment for schistosomiasis in 2018 (WHO, 2020), while estimates predict that 218 million additional patients living in areas at risk of transmission should be treated (WHO, 2020).

The epigenome of schistosomes and their intermediate hosts is very plastic. We and others have shown that the chromatin structure of the parasites undergoes dramatic changes during development from the infecting larvae forms to the adults that mate in the vertebrate host (Roquis et al., 2018). Epigenetic plasticity was also demonstrated in the vector snail and intermediate host *Biomphalaria glabrata* (Geyer et al., 2017) and related to its capacity to resist infections (Knight et al., 2016) (reviewed by de Carvalho Augusto et al., 2019). Epigenetic mechanism play an important role in the trained immune response in vertebrates and it is not surprising that they might also be involved in the establishment of immune memory in invertebrates (Netea et al., 2016; Melillo et al., 2018). It is interesting to note that pathogens have evolved mechanisms to target the epigenetic eukaryotic command centers (Hanford et al., 2021).

Taken together it is conceivable that epigenetic modifications play an important role in the development and the immune response of vector snails. The problem is that we must rely on nature providing us with occasions to study phenotypic effects of rare and uncontrolled epimutations to address this question. It would be desirable to introduce epimutation at specific target loci to understand their phenotypic effects. Epigenetic editing exits for vertebrate host exists but not for snail and snail-borne diseases. Here we addressed this issue and developed a technique for the introduction of epimutations in the freshwater snail *B. glabrata,* intermediate host of the human parasite *Schistosoma mansoni*.

DNA methylation is an epigenetic mark that has been shown to play an important role in the control of gene expression, in genome imprinting, transposon silencing, and X-chromosome inactivation in mammals (Jones, 2012). In vertebrates, genomes are broadly and deeply methylated, besides, partial, and local hypomethylation is found at the gene promoters, where hypermethylation induces gene silencing (Esteller, 2002). In contrast, in invertebrates, DNA methylation tends to occur at moderate levels, and is mostly found in the gene bodies (gene body methylation, GBM) (Keller et al., 2016). It was hypothesized that GBM in invertebrates is associated with active transcription since it preferentially occurs in highly transcribed genes (Zemach et al., 2010; Sarda et al., 2012). However, no mechanistic link to gene expression has been demonstrated for such type of DNA methylation and its function remains elusive for the time being.

Consequently, it is essential to develop approaches that allow us to study GBM and its relationship with gene expression. Currently, the method of choice for the modification of GBM in invertebrates is chemical treatment, but this approach does not allow us to target specific genome loci (Chiappinelli et al., 2015) and an alternative is to repurpose the sequence specificity of the Cas9 enzyme to direct DNA methylation machinery to a desired location. The CRISPR-Cas type II system from *Streptococcus pyogenes* that consists of the nuclease Cas9 and a single guide RNA (sgRNA) (Wu et al., 2014) is the most used genetic editing tool at present. Both can form a ribonucleoprotein complex (RNP) that recognizes and binds to a specific genomic region containing the protospacer adjacent motif (PAM). The targeting of this region is due to the sgRNA that includes a sequence of 20 nucleotides upstream the PAM sequence, an NGG motif for the system coming from *S. pyogenes*. The sgRNA sequence will match the genomic sequence that is located upstream the PAM sequence, and Cas9 nuclease will cleave both strands of the genomic DNA, leaving a DNA double stranded break (DSB) generally between the third and fourth nucleotides upstream PAM (Shan et al., 2020). Then, the DSB will be repaired by non-homologous end-joining (NHEJ) that can introduce deletion or insertion of one or several desoxyribonucleotides. Alternatively, homology-directed repair (HDR), that is less efficient but more precise for introducing a DNA template, can occur (Zaboikin et al., 2017).

Beyond gene editing, the sequence recognition capacity of Cas9 has been adapted to perform epigenome editing such as targeted DNA methylation. Cas9 without nuclease activity, deadCas9 (dCas9), and an sgRNA allowing the modifications of DNA methylation at precise gene loci when fused to the catalytic domain of the DNA methyltranferase DNMT3A which is the most active isoform of a *de novo* DNA methyltranferase in eukaryotes, it methylates CpG sites *in vivo* and *in vitro* (Oka et al., 2006).

Methylome engineering tools have been mainly applied to various models such as mice or mammal cells in culture, and multiple reviews discussed the advancements on epigenome editing in these models (Holtzman and Gersbach, 2018; Lei et al., 2018; Plummer et al., 2018). In a recent work it was demonstrated that the dCas9-DNMT3A construct increases CpG methylation and silences gene expression in mammalian cells. The methylation level at the targeted loci varies from 25 to 55% after 7 days in transfected HEK293 cells and cultured cells lost the construct 10 days after the transfection with the dCas9-DNMT3A plasmid (Vojta et al., 2016). In contrast, *in vivo* transfection in “non-model” species is a technique that has proofed challenging, and few studies have been conducted in invertebrates apart from the two invertebrate models *Drosophila* (Lin et al., 2015) and *Bombyx mori* (Liu et al., 2019). In the present work we used the methylome engineering system dCas9-SunTag-DNMT3A in a mollusk, the freshwater snail *B. glabrata* since it is one of the intermediate hosts of the human parasite that causes schistosomiasis, the second most important parasitic disease after malaria.

SunTag is a protein scaffold containing multiple epitopes able to recruit effector domains (Tanenbaum et al., 2014). The SunTag system consists of a repeating peptide array that can recruit multiple copies of an antibody-fusion protein (scFv) associated with DNMT3A, which increases the local concentration of DNMT3A improving the targeted *in vitro* DNA methylation. Besides, the SunTag scaffold amplifies the intensity of the fluorescent signal due to the multiple associated GFP, facilitating the tracking of protein expression (Huang et al., 2017). dCas9-SunTag-DNMT3A has been successfully applied to target the HOXA5 locus in human kidney cells (HEK293T) where it showed a highly efficient increase in promoter methylation (60–95%) inducing a HOXA5 mRNA repression (Huang et al., 2017).

The snail *B. glabrata* is indispensable for the life cycle of the Schistosome parasite and DNA methylation machinery of the snail has been shown to be affected by the parasite infection. The parasite *S. mansoni* can modify the transcript abundance of the DNA methylation machinery enzymes of the snail *B. glabrata* during infection, suggesting that the DNA methylation of the snail could have a role in their interaction with the parasite (Geyer et al., 2017). Furthermore, the parasite modifies the DNA methylation of certain genes in the snail, which alters its gene expression (Knight et al., 2016). However, the causal relationship between DNA methylation and plasticity of gene expression in the snail and in invertebrates remains enigmatic, and methylome engineering tools are indispensable to establish clear causal relations between DNA methylation and gene expression.

We select a homologue of a homeobox gene to test the methylome engineering tool since it is involved in the morphogenesis of the development of the nervous system during embryogenesis in other mollusks used as biological models in developmental biology, such as *Haliotis asinine* (Hinman et al., 2003) which implies that this gene is expressed during embryogenesis which could in the future make it possible to verify whether the expression of genes is altered by modifying its methylation of DNA.

We have microinjected two plasmids coding for the dCas9-SunTag-DNMT3A system and an sgRNA and we obtained fluorescent-positive snail embryos after *in vivo* transfection. Analysis of positive embryos demonstrated a successful increase in targeted GBM at CpG sites near the PAM motif of the sgRNA in the homeobox gene Nkx2.5 in the snail *B. glabrata*.

## Materials and Methods

### Ethics Statement and Plasmid Transformation

*B. glabrata* albino Brazilian strain (*Bg*BRE) was used in this study. Snails were maintained at the IHPE laboratory facilities; 8 groups of 10 snails were kept in separate aquariums and fed with lettuce *ad libitum*, polystyrene pieces were introduced to the aquariums since the snails use it to lay their egg patches*.* The Direction Départementale de la Cohésion Sociale et de la Protection des Populations (DDSCPP) provided the permit N°C66-136-01 to IHPE for experiments on animals. Housing, breeding, and animal care were done following the national ethical requirements. Plasmids were constructed as in Huang et al., 2017. One Shot™ TOP10 Chemically Competent *Escherichia coli* cells (Invitrogen, Cat. num. C404004) were transformed with the plasmids dCas9-SunTag and scFv-DNMT3-GFP. Subsequently, PCR was performed (Table 3) to confirm the transformation with the dCas9 and DNMT3A sequence inserts. Primers were elaborated in the web-interface Primer3Plus.

### Selection of Candidate Genes

To identify suitable target genes for targeted DNA methylation epimutagenesis the following criteria were established:

1) Candidate genes were selected from known homeobox genes in *Haliotis asinine* (Hinman et al., 2003), homologues of genes in *B. glabrata* were identified by BLAST searches in the NCBI nucleotide collection database. Homologues of homeobox genes were used to be sure they were expressed during embryogenesis.
2) The DNA methylation level of the selected genes was visually examined using our reference methylome (https://zenodo.org/record/4277533) in the IGV-Integrative Genomics Viewer (http://software.broadinstitute.org/software/igv). The criteria to select a candidate gene was that it must be unmethylated.
3) The tissue expression of each gene was observed in a local RNA-Seq database to select those genes that are expressed in most tissues or at least in tissues that are easily accessible and, therefore, easy to manipulate (DNA/RNA extraction).
4) Local adult transcriptome data and a multi-stage transcriptome of *B. glabrata* (Kenny et al., 2016) was used to align adult transcriptome reads with multi-embryo stage transcriptome reads, and we used HTseq-count to verify if genes were expressed at the embryo phase.

### Elaboration of gRNAs

The homeobox Nkx 2.5-like gene was selected, a homologue of the *Has-Hox4* of the gastropod *Haliotis asinine* (GenBank: AAK11240.1), whose expression is restricted to the forming pleural ganglia and nerve cords, suggesting a role in the nervous system development (Iijima et al., 2006). Three gRNAs targeting DNA methylation canyon on the target locus: LGUN_random_Scaffold680: 40871-42922 on *B. glabrata* gene database (https://legacy.www.vectorbase.org) were elaborated by a software for designing CRISPR/Cas9 guide RNAs called CHOPCHOP (Montague et al., 2014). The off-target was also predicted against *B. glabrata* genome by CHOPCHOP as previously described (Coelho et al., 2020) and following considerations for dCas9-DNMT3 system described in (Brezgin et al., 2019). The three sgRNAs were designed in the CUFF52452. Oligonucleotides for the three sgRNAs (Table 1) targeting the gene were ordered from Sigma-Aldrich and Synthego.

**TABLE 1:** sgRNAs designed to target the homeobox 2.5 Nkx of *B. glabrata*.

ID	RNA sequence [PAM]	DNA sequence [PAM]	Location
sgRNA1	GGA​GUA​GUG​AGG​CUG​CUG​UG[AGG]	GGA​GTA​GTG​AGG​CTG​CTG​TG[AGG]	LGUN_random_Scaffold680:42773-42795
sgRNA2	AAC​GAC​GGU​UCA​AGC​AGC​AG[AGG]	AAC​GAC​GGT​TCA​AGC​AGC​AG[AGG]	LGUN_random_Scaffold680:42533-42555
sgRNA3	CUC​UGA​AAC​UAU​UUG​UUU​UC[AGG]	CTC​TGA​AAC​TAG​TTG​TTT​TC[AGG]	LGUN_random_Scaffold680:41352-41372

### Efficiency of the *in vitro* DNA Cleavage Activity of the Cas9-sgRNA Complex

To evaluate the cleavage efficiency of the Cas9-sgRNA complex, a PCR was performed to amplify the targeted region of the sgRNA. Forward and reverse primers were designed to amplify a region spanning the sgRNA target in the middle of the amplified sequence to be able to distinguish cleaved fragments from non-cleaved fragment in the electrophoresis gel. The PCR amplification was set as follows: 95°C for 1 min, followed by 30 cycles of 95°C for 30 s, Tm specific of the couple of primers for 30 s, 72°C for 2 min, and last 72°C for 10 min. The primers for each gene region are detailed in Table 2.

**TABLE 2:** Primers designed to verify sgRNA cleavage efficiency.

ID	sgRNA	Sequence	Tm (°C)	Amplicon length
HP2.5-1 For	sgRNA 1	GTC​GCT​GCT​TCA​GCA​AAG​TAC	51	315 bp
HP2.5-1 Rev		GTA​AAC​TAA​CTT​GCA​CTC​AGC		
HP2.5-2 For	sgRNA 2	TCT​TTC​CTT​TGT​CTT​TCT​CGC​T	52	201 bp
HP2.5-2 Rev		GCA​CTC​AGC​TTT​CTT​CAC​TTC​A		
HP2.5-3 For	sgRNA 3	GTG​TGT​GTG​TCG​TTT​GAC​AAA​G	51	296 bp
HP2.5-3 Rev		CGA​CTG​TCT​ACA​CAA​TTC​TGT​G		

The DNA cleavage activity of the previously amplified PCR fragment was assayed *in vitro* with Cas9-sgRNA complex as previously described (Mehravar et al., 2019). The cleavage reaction was performed by mixing 1 µl of sgRNA (1 μg/μl), 3 µl 10X NEBuffer 3.1 (New England Biolabs, B7203S), 3 µl Cas9 recombinant protein (200 ng/μl), 1 µl of the PCR product of the targeted gene, and the volume was completed to 30 µl with nuclease-free water. The reaction was incubated at 37°C for 2 h, then 1 µl of proteinase K (20 mg/ml) was added to the reaction and a final incubation was done during 10 min at room temperature. Reactions with all components except for sgRNA were used as negative controls. Recombinant protein Sp-Cas9-NLS-GFP-NLS was a generous gift of Jean Paul Concordet from the INSERM U1154, CNRS UMR7196, Structure and Instability of Genomes, Sorbonne Universités, Museum National d’Histoire Naturelle in Paris, France. The amplicon digestion on the target site after sgRNA transfection was revealed by 2% agarose electrophoresis gel stained with Midori Green Advanced (Nippon Genetics Europe, catalog number MG04).

### Microinjection Tests

The linear polyethylenimine derivative, *in vivo*-jetPEI™ (Polyplus Transfection, France) was used for a survival assay with five early stages of embryonic development. Two-cell, 8-cell, morula, blastula, and gastrula stages were determined based on the descriptions and embryo illustrations in (Camey and Verdonk, 1969). Egg patches were collected from the aquarium and sorted under the microscope to identify the five developmental stages, 50 embryos per developmental stage were used to test *in vivo* jetPEI reagent. Microinjection of embryo yolk sacs with *in vivo* jetPEI reagent and the two plasmids coding for dCas9-SunTag and DNMT3-GFP has been performed. A pre-pulled glass micropipette of 1 mm of diameter and a tip of ∼200 µm of diameter was attached to a programmable nanoliter injector (Nanoject III, Drummond Scientific) to carry out microinjections in the embryos’ yolks under a stereoscopic microscope. The parameters used in the nanoliter injector were: injection volume 30 nanoliters and speed of the injection of 20 nanoliters per second.

Glucose solution and *in vivo* jetPEI were equilibrated at room temperature. The scFv-DNMT3A-GFP plasmid concentration was 78 ng/μl and the dCas9-Suntag plasmid concentration was 88 ng/μl. A total of 21 µl of each plasmid (42 µl of total volume = 3.5 µg total amount of plasmid DNA) were added to 21 µl of 10% glucose in a tube (tube A), and in another tube (tube B), the quantity of *in vivo* jetPEI required for the amount of DNA was added (1 µl) to 21 µl of 5% glucose. After 15 min of incubation, tube A and tube B were mixed giving a total of 91 µl of microinjection volume with a final concentration of plasmid DNA of 41 ng/µl that was used as a microinjection transfection solution, this volume was enough to microinject approximately 250 embryos. After our survival assay, we identified that the microinjection was less lethal at the gastrula stage compared to the other early stages. We therefore proceed to transfection by microinjection at this stage.

Then, we needed to test transfection efficiency, therefore 400 embryos at the gastrula stage were transfected. Once we validated transfection efficiency by RT-qPCR in 16 fluorescence positive transfected embryos from 400 microinjected (4%), we therefore microinjected 250 embryos at the gastrula stage with the microinjection transfection solution and then selected and separated the fluorescent embryos 72 h after transfection. Another solution was prepared with 21 µl of glucose 5% and 0.5 µl of *in vivo* jetPEI and was used to microinject controls that consist of another 100 embryos at the gastrula stage. Hence, the mixture of *in vivo* jetPEI (0.5 µl), 5% glucose (10 µl), and active sgRNA3 (20 ng in 10 µl) was injected into the 10 fluorescent positive embryos and into 10 control embryos. Three days after sgRNA3 transfection, newly hatched snails were collected and stored at −80°C for DNA extractions and subsequent amplicon sequencing after bisulfite treatment.

### Confocal Microscopy

Individual fluorescent larvae were washed three times in PBS solution (Sigma-Aldrich, catalog number: P4417) and then fixed with 500 µl of 4% paraformaldehyde (PAF) (Sigma-Aldrich, catalog number: 158127) for 2 h at room temperature. Single larvae were put on a microscope slide with 2 drops of the fluorescence mounting medium Dako (Agilent Technologies, catalog number: S3023) and the sample was covered with a coverslip. Slides mounted were stored overnight at 4°C in the dark to preserve fluorescent signal. Slides were examined under an epifluorescence confocal microscope LSM700 (Zeiss). This protocol was adapted from a previously published protocol (Duval et al., 2013; Galinier et al., 2013).

### *B. glabrata* Embryo DNA Isolation

A DNA purification protocol used for zebrafish embryos was adapted to *B. glabrata* hatched snails (Jing, 2012). Briefly, 40 µl of lysis buffer containing 9.4 ml of 10 mM Tris-HCL (pH 8.3), 50 mM KCl buffer, 300 µl of NP40 (10% stock), and 300 µl of Tween 20 (10% stock) was applied to single embryos. Incubation at 98°C for 10 min to lyse cells was applied and then 5 µl of proteinase K (10 mg/ml) were added to each embryo. Incubation at 55°C for 2 h was done followed by a final incubation of 5 min at 98°C to heat kill proteinase K. DNA was directly used for PCR or bisulfite conversion. PCR mix, template DNA, and primers (Table 2) were set as follows: 94°C for 2 min, 5 cycles of 94°C for 1 min, 46°C for 2 min, and 72°C for 3 min, followed by 25 cycles of 94°C for 30 s, 51°C for 2 min, and 72°C for 1:30 min and finally 72°C for 10 min. Then a 2%—agarose gel was elaborated to run PCR reactions and identify if the target gene was amplified.

### Isolation of Poly (A+) mRNA From *B. glabrata* Embryos and Reverse Transcription

For messenger RNA isolation, individual trochophore larvae were collected in RNAse-free tubes with 100 µl of lysis buffer of the Dynabeads mRNA DIRECT Micro Purification Kit (Cat. Num. 6102, Invitrogen) and stored at −80°C. mRNA was purified using the Dynabeads mRNA DIRECT Micro Purification Kit according to the manufacturer’s instructions. This method is based on base-pairing between the poly-A residues at the 3′ end of the mRNA and the oligo-dT_25_ residues covalently couple to the surface of the paramagnetic beads. Reverse transcription to first strand cDNA was done using Maxima H Minus First Strand cDNA Synthesis Kit with dsDNase (Cat. Num. K1682, ThermoFisher, Scientific). A DNase mixture (dsDNase, 10X dsDNase Buffer and water nuclease-free) was directly added to the bead-trapped mRNA, and then the reaction was done in a final volume of 20 µl (10 mM dNTP Mix, water nuclease-free, 5X RT Buffer, and Maxima H Minus Enzyme Mix). An initial incubation step for 5 min at 50°C was done. Then the reaction was incubated for 10 min at 25°C followed by 15 min at 50°C. To terminate the reaction, an incubation at 85°C for 5 min was done.

### RT-qPCR to Check for Reporter Gene Transcription

Real-time qPCR analyses were done on cDNA obtained from 8 controls and 16 transfected embryos that were those that showed fluorescence 72 h after plasmids transfection. Low amounts of cDNA were obtained per larva (≤0.5 ng/µl); therefore, the cDNA of each larva was split in 8 reactions, 2 RT positive replicates and 2 RT negative replicates per target gene and per housekeeping gene (28S). Before qPCR, cDNA was pre-amplified for each couple of primers (Table 3) when still attached to Dynabeads as follows: 95°C for 5 min, 4 cycles of 95°C for 5 s, 60°C for 30 s and 72°C for 30 s, then 72°C for 5 min, and a final incubation at 94°C for 2 min to separate cDNA from Dynabeads. Then qPCR was done using the LightCycler 480 System (Roche) in a 17.5 µl final volume comprising 10 µl of No Rox SYBR Master Mix blue dTTP (Takyon), 3.5 µl of ultrapure MilliQ water, and 1 µl of each primer (Table 5) at a concentration of 10 µM (final concentration of primers was 500 nM) and the pre-amplified cDNA (∼0.5 ng). To ensure the absence of genomic DNA contamination, a reaction with all reagents except reverse transcriptase (RT) was done per samples (two replicates per sample) as an RT negative control.

**TABLE 3:** Primers designed to check for plasmid gene transcription.

ID	Sequence	Amplicon thlength
28S for	GCT​GGC​ACG​ACC​GCT​CCT​TT	100 bp
28S rev	TTT​GAA​CCT​CGC​GAC​CCG​GC	
GFP for	GAA​TTA​GAT​GGT​GAT​GTT​AAT​GGG	254 bp
GFP rev	TTG​AAA​GAT​ATA​GTG​CGT​TCC​T	
DNMT3A-1 for	TGA​TTG​ATG​CCA​AAG​AAG​TGT​C	217 bp
DNMT3A-1 rev	AAC​ACA​GGA​AAA​TGC​TGG​TCT​T	
BFP-2 for	CAA​GGA​GGC​CAA​CAA​CGA​GA	80 bp
BFP-2 rev	CCA​GTT​TGC​TAG​GGA​GGT​CG	
dCas9-2 for	AAA​GAA​GGA​CTG​GGA​CCC​TAA​G	233 bp
dCas9-2 rev	CAG​AAA​GTC​GAT​GGG​ATT​CTT​C	

The cycling program had a denaturation step at 95°C for 2 min, 40 cycles of amplification (denaturation at 95°C for 10 s, annealing at 58°C for 20 s, and elongation at 72°C for 30 s), and a final elongation step at 72°C for 5 min. PCR experiments were performed in duplicate (technical replicates). The mean value of Ct and melting curves was checked using the LightCycler 480 Software release 1.5.0.

### Bisulfite Conversion

Bisulfite conversion was done as previously described in our preprint (Luviano et al., 2021). A total of 300 ng of DNA extracted from adult snails was used as a control and 20 ng from embryo snails (10 control and 10 transfected) was denatured by adding 2 µl of ribonucleic acid transfer from baker’s yeast (*S. cerevisiae*) and 2.2 µl of 3 M NaOH, and by incubating at 42°C for 20 min. Then 240 µl of fresh prepared sodium bisulfite solution (5.41 g sodium metabisulfite in 7 ml of distilled water and 0.5 ml of diluted hydroquinone) (0.022 g/10 ml) was added to the denatured DNA samples. An incubation in the dark was done during 4 h at 55°C. Then 200 µl of distilled water was added to the samples, and the total volume was transferred to an Amicon column (UFC501024, Millipore), a centrifugation was done at 12,000 g during 5 min. The column was washed 3 times with 350 µl of distilled water and centrifugation at 12,000 g for 5 min was done each time. Following this, 350 µl of 0.1 M NaOH was added to the DNA in the Amicon column and a centrifugation at 12,000 g during 5 min, subsequently 350 µl of distilled water was added and a centrifugation at 12,000 g for 5 min was done. Twenty microliters of 10 mM TRIS/Cl pH 7.5-8.0 was added to the DNA in the Amicon column and incubation at room temperature for 5 min was done. Finally, the DNA was collected by centrifugation at 1000 g for 3 min. DNA was stocked at −80°C for further experiments.

### Nested PCR on Bisulfite Converted gDNA

The initial PCR amplification was done with 5 μl of the bisulfite converted gDNA as a template with external primers (Table 4) set as follows: 94°C for 2 min, 5 cycles of 94°C for 1 min, 46°C for 2 min, and 72°C for 3 min, followed by 25 cycles of 94°C for 30 s, 46°C for 2 min, 72°C for 1:30 min, and finally 72°C for 10 min. The nested PCR was performed on 2.5 μl of the first PCR product using the internal primer set in the same condition as for the first PCR except for the annealing temperature which was increased to 50°C. The subsequent PCR reaction was performed in 25 µl using 1.25 units of GoTaq DNA polymerase (Promega), dNTPs at 0.4 µM for each deoxynucleotide, and primers at 0.4 µM. PCR products were separated by electrophoresis through 2% agarose gels to check for the specific amplification of each target gene. The house-keeping gene *Bg14.3.3* (BGLB005695, Scaffold 1582:42425–42875) was used as a control, this gene was previously shown to be highly methylated (Geyer et al., 2017), which allows us to evaluate the efficiency of the bisulfite conversion. Bisulfite PCR products were sequenced by Sanger sequencing (Genoscreen, Lille, France). Sequence chromatograms were analyzed as previously described to calculate cytosine to thymine conversion by measuring cytosine and thymine height peaks. This provides the degree of methylation of the targeted sequences (Jiang et al., 2010).

**TABLE 4:** Primers designed to check for efficiency of bisulfite conversion.

ID	Type of primer	Sequence	Tm	Amplicon length
Bg14.3.3 BS 5	External	GAt​TGA​AtT​TGA​AGG​TAA​ATT​AAt​AtA​AGA	58.1	605 bp
Bg14.3.3 BS 5	External	aAT​aTC​CAC​Aaa​aTA​AaA​TTa​TCA​C	55.4	
Bg14.3.3 BS 1	Internal	tTG​TtT​GTT​GtT​TAt​AAA​AAT​GTT​GTG	59.4	478 bp
Bg14.3.3 BS 1	Internal	CAC​TaA​TAa​CCT​CAT​CAA​Aaa​CCT​CTT	62.6	
HP2.5-II For	External	GAA​GTt​TTG​GAG​GTt​AAA​AAt​TTT​Gt	60.6	838 bp
HP2.5-II Rev	External	aAC​TCA​aCT​CAa​ATC​AaT​AAA​TCC​AC	61.4	
HP2.5-III For	Internal	TGT​GAG​AAt​tAt​TtT​ATT​AAG​TTG​T	56.3	458 bp
HP2.5-III Rev	Internal	TaT​TTT​CAa​aAC​ATA​CCT​CTT​TaA​C	57	

Note: Bisulfite conversion of C to T in forward primers is indicated by lowercase “t” and in reverse primers G to A conversion is indicated by lowercase “a”.

### Amplicon Sequencing Library Construction Obtained from PCR Performed on Bisulfite Converted gDNA

For the amplicon sequencing library, the bisulfite converted DNA of 10 control and 10 transfected snails was used as a template for a bisulfite PCR with the external bisulfite primers HP2.5-II (Table 4) and a nested PCR was performed with the internal bisulfite primers containing overhangs for subsequent indexing. For the HP2.5-III forward primer the overhang was: 5′TCGTCGGCAGCGTCAGATGTGTATAAGAGACAGNN- [primer sequence]3′ and for the HP2.5-III reverse primer: 5′GTCTCGTGGGCTCGGAGATGTGTATAAGAGACAGNN- [primer sequence]3′. The nested PCR was purified with 0.9X AMPure XP beads (Beckman Coulter) to be indexed. Dual indexing was done using the Illumina Nextera XT Index Kit following manufacturer’s instructions. After indexing, bead purification was done in amplified libraries with 0.9X AMPure beads and quantification of each indexed sample was done in Qubit fluorometer with the Qubit dsDNA HS (High Sensitivity) Assay Kit (Thermofisher Scientific). Then each sample was normalized to equal nanomolar concentration and then pooled. One µL of the pooled libraries was analyzed on a Bioanalyzer (Agilent) and after checking broad size distribution, the libraries were sequenced in MiSeq system (Illumina) at the Bio-Environment NGS Platform at the University of Perpignan.

### Bioinformatics and Statistics

Raw reads were trimmed using Trim galore default parameters to remove Illumina adapters, then trimmed reads were mapped to the reference genome of *B. glabrata* with the alignment algorithm BWA-MEM that map long reads (>100 pb) to verify that the targeted gene was sequenced. After that, trimmed reads were used as input in the command line version of the software pipeline CRISPResso2 available at https://github.com/pinellolab/CRISPResso2. This pipeline allows us to align reads to a reference sequence, and to obtain conversion of target bases around the sgRNA and in the entire amplicon which allows us to calculate the conversion percentage of cytosines transformed to thymines in the target CpG sites (Clement et al., 2019). Nucleotide frequency of the entire amplicon was obtained from this pipeline and CpG sites were searched manually to calculate the cytosine to thymine conversion. The normality of the data distribution was tested with the Shapiro-Wilk normality test. For comparing CpG%, between controls and transfected samples, a Mann-Whitney test was performed. To test whether there was a significant association between transfection and methylation increasing, we performed Fisher’s exact test. The 2 × 2 contingency table used to apply this test contained two categorical variables, group (Transfected and Control) and the methylation status (Methylated and Not methylated). In the control group, there were 0 snails methylated and 9 not methylated. In the case of the transfected group, there were five snails methylated and five not methylated.

### Visual Support

Detailed descriptions of the method with accompanying video in French and English are available at Zenodo (https://doi.org/10.5281/zenodo.4549023, https://doi.org/10.5281/zenodo.4549069).

## Results

### Microinjection With *in vivo* jetPEI Produces Superior Survival Rates at the Gastrula Stage and Plasmid Expression is Observed 72 h After Transfection

Microinjection was done at five early stages of embryonic development (Table 5) with an *in vivo* transfection reagent (*in vivo*-jetPEI). The survival percentage was very low in the embryo snails transfected before the gastrula stage (0–10%), while from the gastrula stage the survival increases up to 50%. Moreover, GFP expression was not observed in any of the embryos injected before the gastrula stage. The GFP expression was observed in the trochophore larvae of embryos transfected at the gastrula stage 72 h post-transfection.

**TABLE 5:** Survival percentages of the transfections done at different embryo developmental stages with the *in vivo* jetPEI reagent.

Time after first cleavage	Stage	Number of injected snails	Number of surviving snails	Survival %
During first cleavage	2-cell stage	50	0	0
160 min	8-cell stage	50	2	4
5 h	Morula stage	50	4	8
18–20 h	Blastula stage	50	5	10
26 h	Gastrula	50	25	50

**FIGURE 1:**
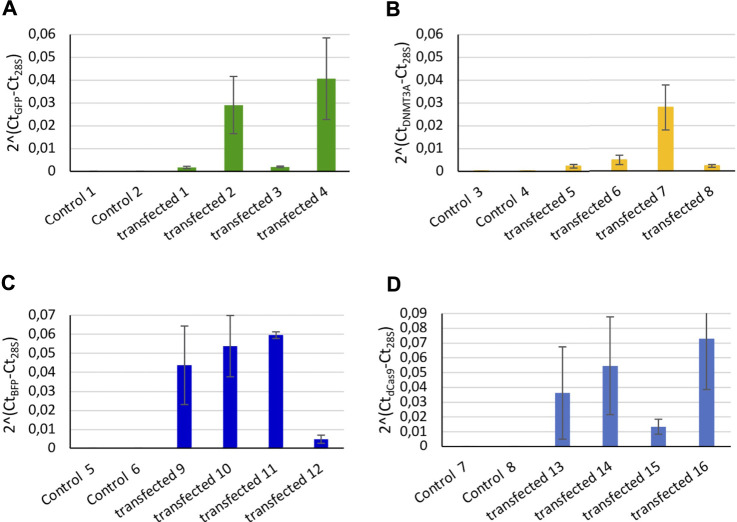
Relative expression of controls (microinjected with only *in vivo* jetPEI reagent) and transfected snails (microinjected with *in vivo* jetPEI and the two plasmids coding for the dCas9-SunTag-DNMT3A system). Relative expression of **(A)** GFP, **(B)** DNMT3A, **(C)** BFP, and **(D)** dCas9 from transfected embryos. Two controls snails were compared to 4 transfected embryos per mRNA, each bar represents two technical replicates of the cDNA extracted from a single larva. Error bars correspond to standard deviation of the two technical replicates from each larva.

The transcription level expression of GFP, DNMT3A, dCas9, and BFP was measured by RT-qPCR in individual trochophore larvae. mRNA of GFP (Figure 1A), BFP (Figure 1B), of the catalytic domain of the DNMT3A (Figure 1C), and of the dCas9 protein (Figure 1D) were detected in transfected snails, while the non-transfected controls did not display the expression of any of the four mRNAs (Figure 1). The negative RT controls do not display the expression of the 4 mRNAs.

**FIGURE 2:**
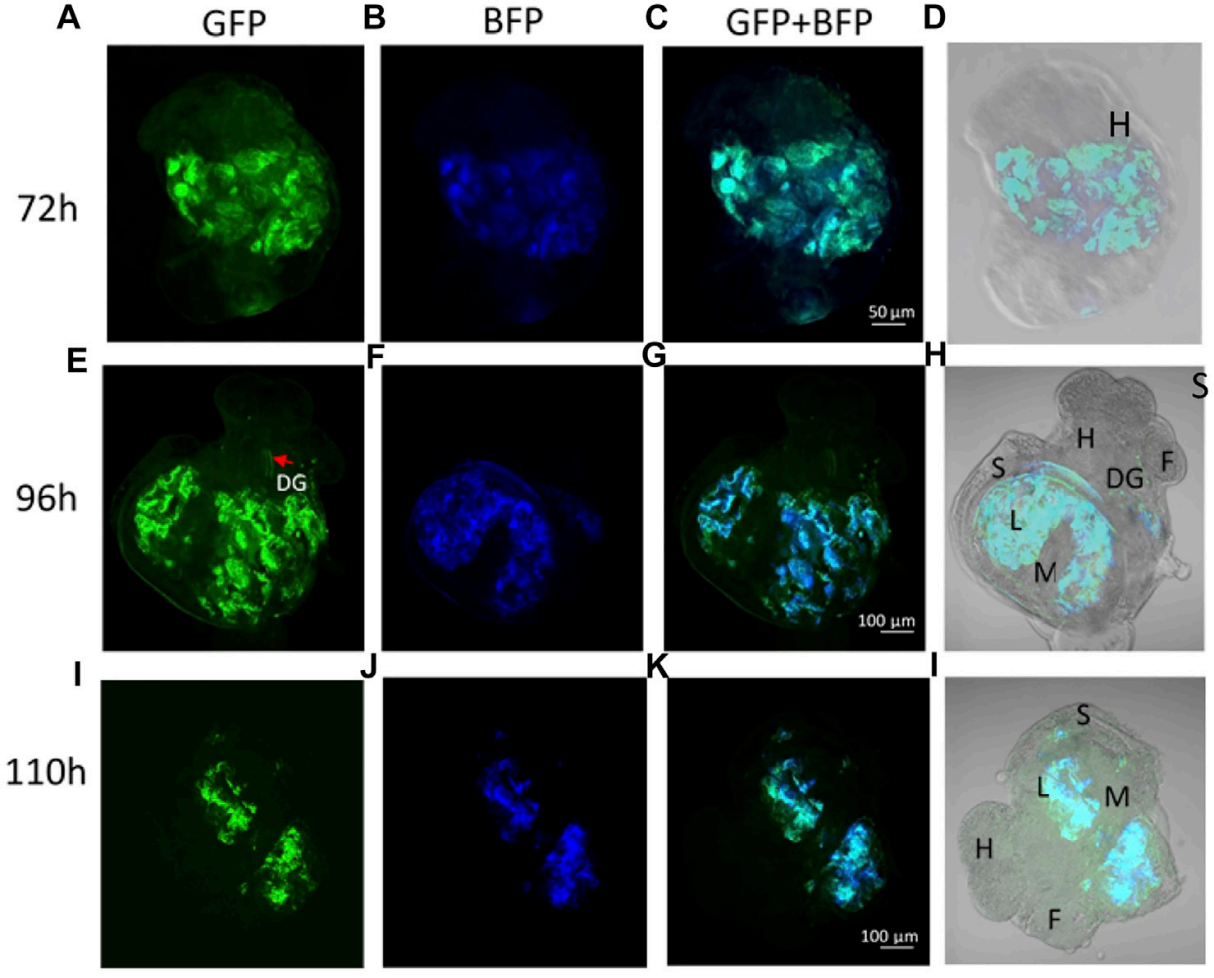
**(A–D)** Trocophore larva 72 h post transfection with dCas9-SunTag-BFP and scFv-DNMT3A plasmids. **(A)** GFP expression, **(B)** BFP expression, and **(C)** GFP and BFP co-localization. **(E–H)** Veliger larva 96 h post transfection. **(I–L)** Snail in hippo stage 110 h post transfection. Anatomical annotations: H-head, F-foot, S-shell, L-lung, M-mantle, DG-digestive gland.

**FIGURE 3:**
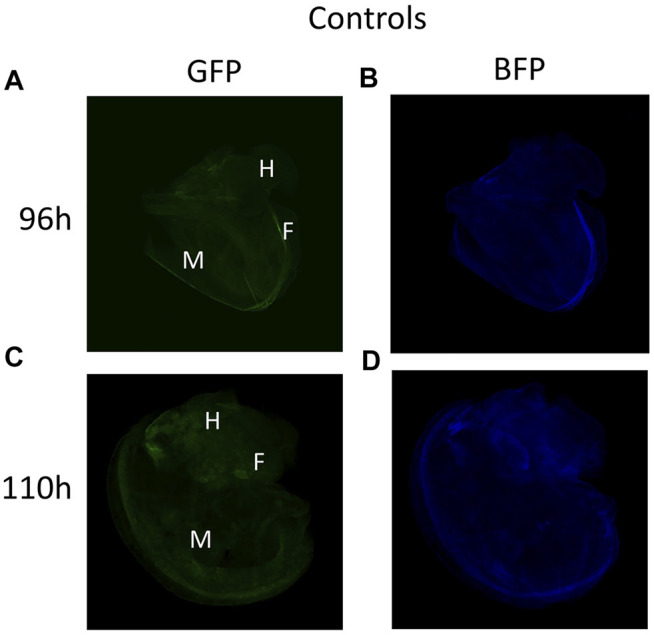
**(A–B)** Veliger larva stage 96 h after microinjection with *in vivo* jetPEI deprived of plasmids and used as a control solution. **(C–D)** Snail in the hippo stage 110 h post-microinjection with control solution.

No fluorescence signal was detected by microscopy until 72 h after transfection. At this stage, the trochophore larvae started to show GFP fluorescence, the reporter gene of the plasmid coding for DNMT3A, and BFP fluorescence for the plasmid coding for dCas9 and the scaffold SunTag (Figures 2A–D). The fluorescence of GFP and BFP continued to be visible (Figures 2E–H) 96 h after transfection and 110 h later, fluorescence was visible but weaker (Figures 2I–L). The fluorescence was no longer visible 168 h post-transfection, demonstrating that the plasmids were expressed transiently in the embryo snail (data not shown). Embryos at the veliger and hippo stage did not show fluorescence expression after the microinjection with a control solution containing the *in vivo*-jetPEI reagent and 5% glucose but deprived of plasmids (Figures 3A–D).

**FIGURE 4:**
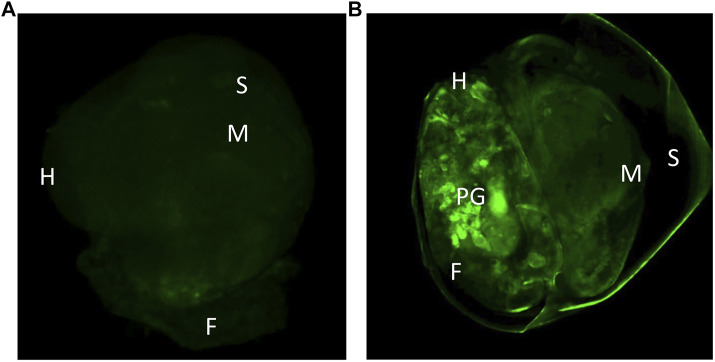
**(A)** Control snail 96 after microinjection with the *in vivo* jetPEI reagent, showing only autofluorescence. **(B)** Snail 96 h after transfection with plasmids dCas9-Suntag and scFv—GFP-DNMT3A. H-head, S-shell, M-mantle, F-foot, PG-pedal ganglia.

The transfected embryos exhibited preferential fluorescence at the ectodermal tissues and at the nervous system. In some photographs we could observe the pedal ganglia in an embryo at the hippo stage (Figure 4). Fluorescence expression was highly mosaic and limited to some cells in the nervous system, therefore mosaic integration was expected.

**FIGURE 5:**
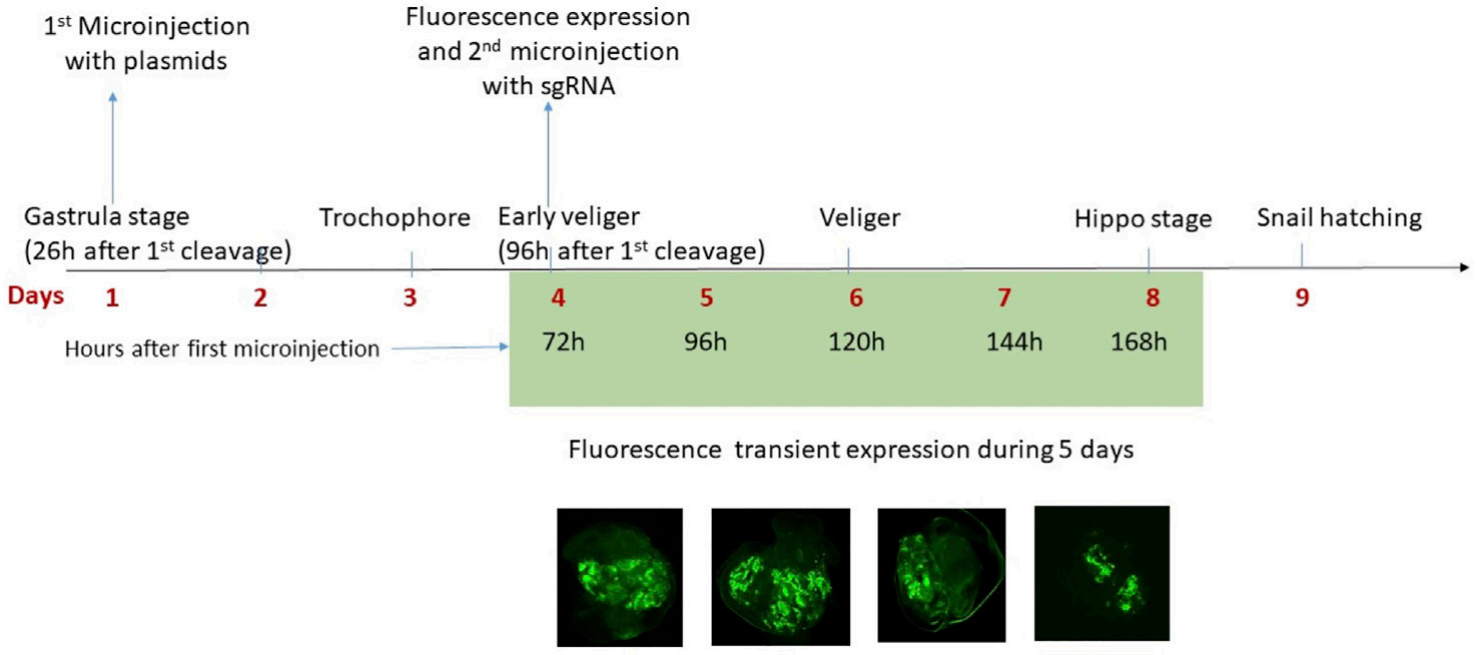
The microinjection was done at the gastrula stage, 26 h after the first cleavage of the zygote. In total, 72 h after the first microinjection the plasmids are expressed in the embryo of *B. glabrata* and in the fluorescent positive embryos a second microinjection with the sgRNA was done. The fluorescence of the reporter genes (GFP and BFP) was observed for 5 days (from Day 4 to Day 8) and then is no longer visible, therefore the expression of reporter genes carried by plasmids is transient. At Day 9, the snails hatch.

In conclusion, transfection in *B. glabrata* is preferable from the gastrula stage since earlier microinjection leads to high mortality rates. Expression of reported genes from plasmid of transfected snails was observed 72 h after transfection and up to 5 days when embryos are at the end of the hippo stage. Approximately 12 h before hatching, the snails lost the fluorescence signal (Figure 5). This dynamic of expression was expected since a transient expression was also observed in transfected human embryonic kidney cells (Huang et al., 2017), we did not select for stable transfection, in our hit-and-run approach, a constitutive expression of the DNMT activity would potentially lead to off-target effects and was not desirable. Total transfection efficiency was 4% (10 fluorescent embryos, from a total of 250 snails).

### Targeted CpG Hypermethylation of 3.4-76% in 50% of Transfected Snails in up to 5 CpG Sites

**FIGURE 6:**
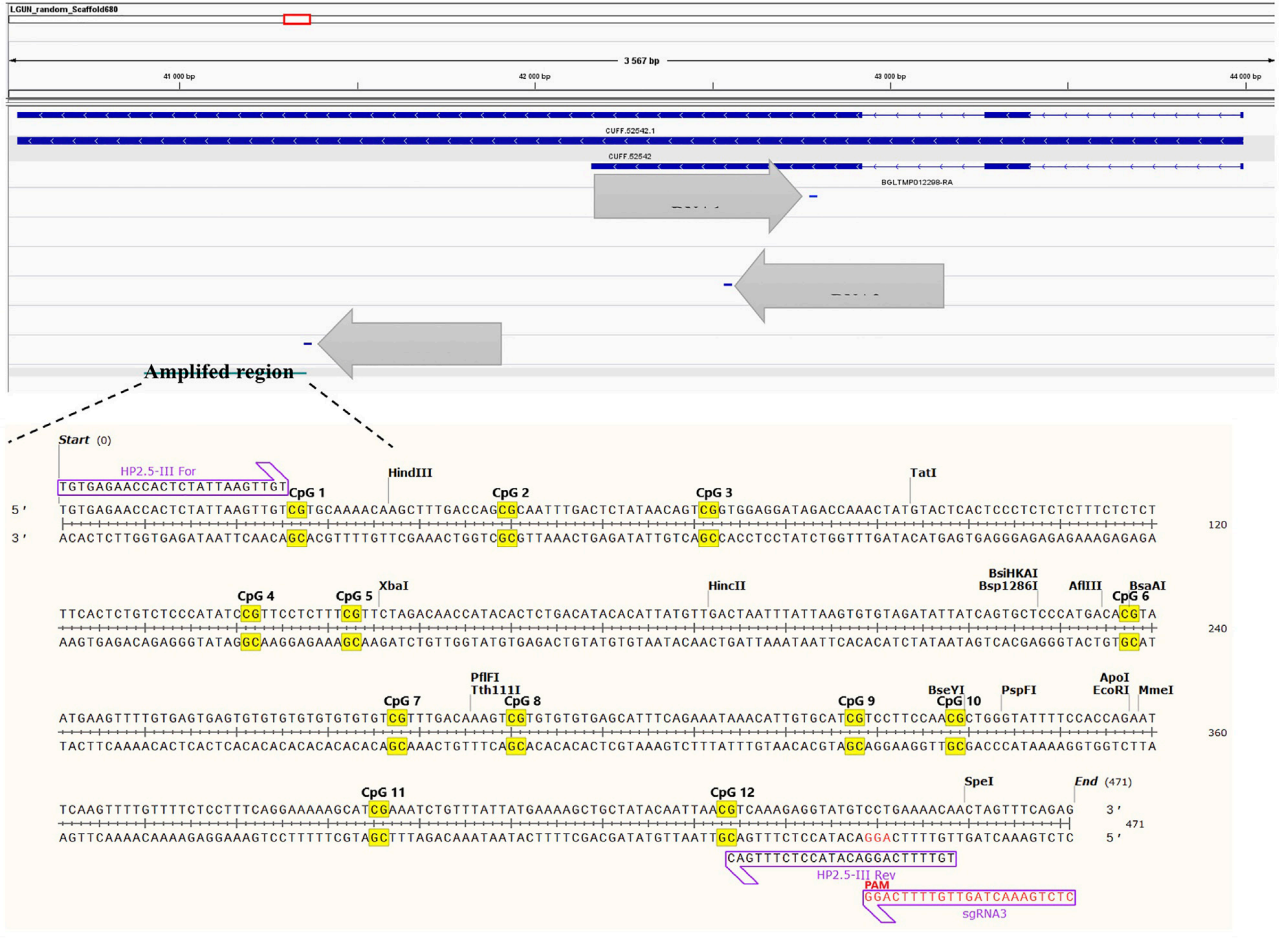
sgRNA position at the targeted locus, whose genomic location is LGUN_random_Scaffold680:40914-41372. The target amplicon is within the gene body and contains 12 CpG sites. The complete sequence of each gRNA is found in the methods section.

**FIGURE 7:**
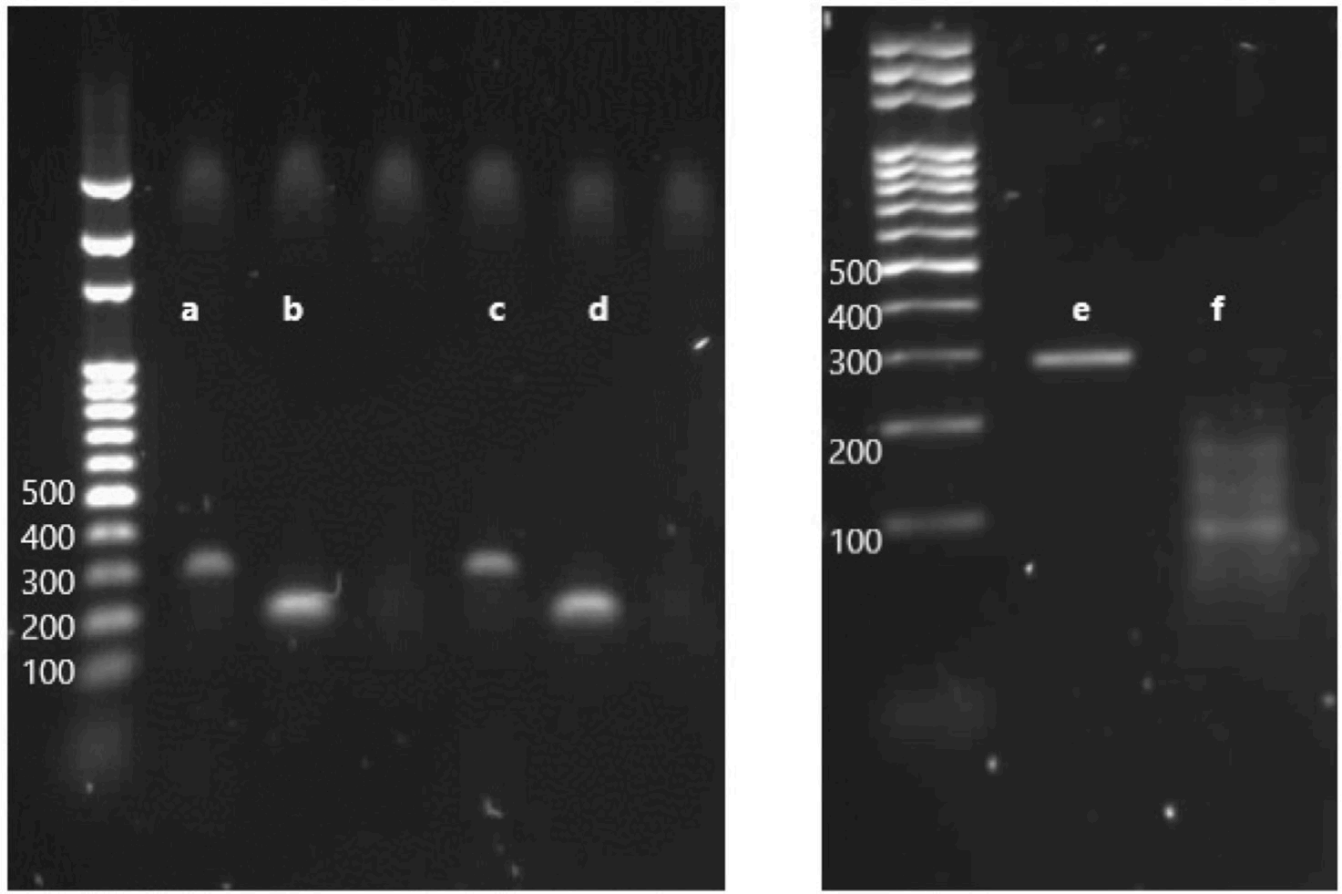
Electrophoresis through an agarose gel to evaluate the *in vitro* efficiency of cleavage by RNP complex on targeted *loci*. **(A)** PCR product with the primers HP2.5-1, amplicon length 315 bp. **(B)** PCR product with the primers HP2.5-2, amplicon length 201 bp. **(C)** PCR product with HP2.5-1 primers incubated with Cas9 and sgRNA1. **(D)** PCR product with HP2.5-2 primers incubated with Cas9 and sgRNA2. **(E)** PCR product of the targeted gene with a couple of primers HP2.5-3, amplicon length 296 bp. **(F)** PCR product of HP2.5-3 primers incubated with Cas9 and sgRNA3, cleaved fragments of the gene, two products—one of ∼188 bp and one of ∼108 bp—corresponding to the size fragments after DSB on the PAM sequence.

After having established a time window in which both dCas9 and DNMT were expressed in the developing snail embryos, injections of guide RNA were performed. To verify the absence of mutations, the targeted *locus* was sequenced by Sanger methodology and to verify methylation level at the target gene, bisulfite Sanger sequencing was done. The target gene was not methylated at any of the 11 CpG sites (data not shown). All three sgRNAs matched to the target sequence and are located at different positions (Figure 6). Cleavage efficiency was assayed *in vitro* and sgRNA3 was shown to be the unique tested guide able to direct Cas9 to cut the targeted *locus* at the PAM sequence (Figure 7). Therefore, positive transfected snails were screened under the microscope at 72 h using fluorescence of the GFP and BFP from the plasmids. At this time, we performed the microinjection of the sgRNA3 in those positive embryos. sgRNA3 was microinjected in the positive fluorescent embryos to maximize dCas9 and sgRNA binding. We then extracted genomic DNA from newly hatched snails and performed the bisulfite conversion. Amplification of the target gene was then performed and analyzed by amplicon bisulfited sequencing (BSAS). This method allowed us to measure accurately the CpG methylation level of 9 controls and 10 transfected snails.

**FIGURE 8:**
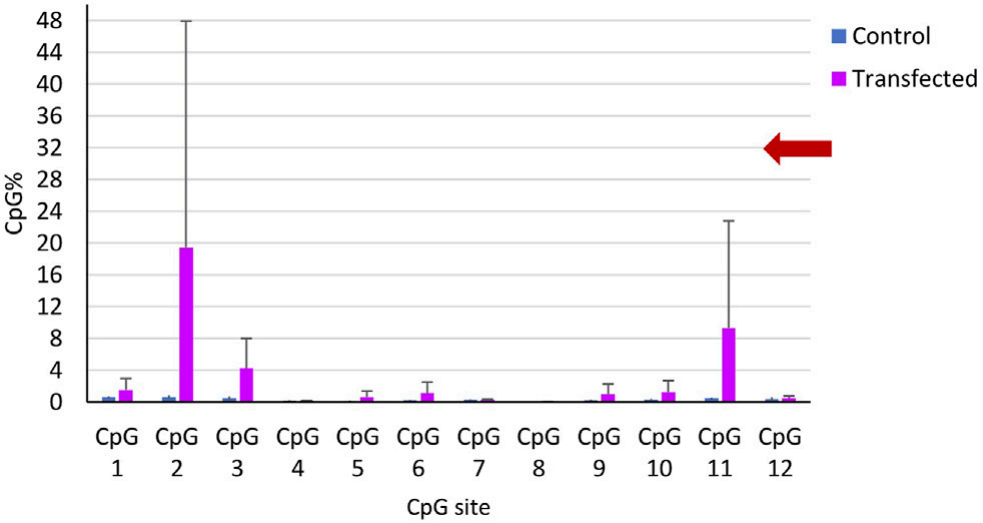
CpG methylation percentage in the 12 CpG sites of the targeted gene along 458 bp. Mean CpG % per CpG site in all controls (blue) and in the transfected snails (magenta). The bars represent the mean CpG methylation percentage per CpG site and the error bars represent the standard error of the mean (SEM).

The two sets of microinjections allowed us to methylate CpG sites in the targeted gene close to the guide RNA. BSAS of the target identify that 4 out of 10 transfected snails showed increased percentage of methylated CpG at the targeted gene (Figure 8), while the controls showed a significantly lower percentage of methylated CpG.

We applied the Mann-Whitney test to compare methylation values between controls and transfected snails, we did not find a significant difference between both groups (*p* > 0.05). We also applied the Fisher exact test in a contingency table of 2 × 2 composed of two variables, group (control or transfected) and the methylation state (methylated or not methylated). We considered as methylated a snail showing at least 1 CpG site with a methylation percentage higher than 2%. From the 9 controls, we observed 0 snails methylated. In the case of the transfected group, we find 5 snails methylated from 10. The difference between the control and transfected group was statistically significant (*p* = 0.03).

The control snails (*n* = 9) displayed ≤1% of CpG methylation in the 12 CpG sites, while 5 of the 10 transfected snails (Transfected 1, 2, 3, 6, and 10) showed an increase of CpG methylation in up to 5 CpG sites (Table 2). One transfected snail (Transfected 6) showed a higher CpG methylation percentage in 5 CpG sites with values ranging from 3.5 to 4.42, while another snail (Transfected 10) showed a weak CpG methylation percentage in only one CpG site (4.4%). The other positive transfected snails showed one CpG site highly methylated (Transfected 1, 2, 3) with values ranging from 12 to 76%. A high heterogeneity was found in the targeted CpG methylation modifications in the transfected snails (Table 6).

**TABLE 6:** CpG methylation results from bisulfite amplicon sequencing CpG sites of the targeted gene BGLB032652 found in the genomic region: LGUN_random_Scaffold680: 40,914-41,372.

Bisulfite amplicon sequencing												
CpG sites	CpG 1	CpG 2	CpG 3	CpG 4	CpG 5	CpG 6	CpG 7	CpG 8	CpG 9	CpG 10	CpG 11	CpG 12
Position on scaffold 680	40927	40950	40972	41042	41054	41138	41178	41191	41228	41239	41293	41334
Control snail 1	0.9	0.4	0.3	0.2	0.3	0.2	0.4	0.0	0.4	0.5	0.5	1.0
Control snail 2	0.7	0.7	0.7	0.2	0.2	0.4	0.3	0.0	0.3	0.4	0.4	0.7
Control snail 3	0.3	1.0	0.5	0.1	0.1	0.3	0.3	0.0	0.4	0.3	0.4	0.3
Control snail 4	0.4	1.0	1.0	0.5	0.2	0.2	0.4	0.0	0.2	0.5	0.5	0.2
Control snail 5	0.7	0.4	0.3	0.1	0.1	0.3	0.2	0.0	0.2	0.3	0.4	0.3
Control snail 6	0.7	0.7	0.4	0.2	0.1	0.2	0.3	0.0	0.2	0.2	0.6	0.3
Control snail 7	0.6	0.4	0.4	0.2	0.1	0.2	0.2	0.0	0.1	0.2	0.5	0.3
Control snail 8	0.7	0.4	0.3	0.1	0.1	0.3	0.3	0.0	0.1	0.3	0.3	0.3
Control snail 9	0.5	0.5	0.3	0.1	0.1	0.2	0.3	0.0	0.1	0.2	0.4	0.3
Transfected snail 1	0.6	0.6	0.6	0.1	0.1	0.2	0.3	0.0	0.2	0.2	**36.3**	1.1
Transfected snail 2	0.7	**76.4**	0.3	0.1	0.1	0.2	0.3	0.0	0.2	0.3	0.4	0.3
Transfected snail 3	0.6	0.4	**11.6**	0.2	**2.1**	0.2	0.3	0.0	0.1	0.2	0.3	0.3
Transfected snail 4	0.6	0.4	0.3	0.1	0.1	0.2	0.4	0.0	0.2	0.2	0.3	0.3
Transfected snail 5	1.3	1.1	0.8	0.1	0.1	0.2	0.3	0.0	0.3	0.3	0.5	0.3
Transfected snail 6	**4.4**	0.4	**4.4**	0.2	0.3	**3.9**	0.3	0.0	**3.5**	**4.2**	0.3	0.2
Transfected snail 7	0.6	0.4	0.3	0.1	0.6	0.3	0.3	0.0	0.2	0.2	0.4	0.2
Transfected snail 8	0.4	0.5	0.3	0.1	0.2	0.3	0.3	0.0	0.2	0.3	0.4	0.3
Transfected snail 9	0.5	0.5	0.4	0.1	0.1	0.2	0.3	0.0	0.2	0.3	0.4	0.3
Transfected snail 10	**4.4**	0.5	0.5	0.2	0.1	0.3	0.3	0.0	0.2	0.4	0.4	1.4

Note: CpG sites showing >2% of DNA methylation were considered as methylated and are highlighted in bold.

The control snails (*n* = 9) displayed ≤1% on the mean CpG methylation of the 12 CpG sites of the targeted gene. The transfected snails that showed increase of CpG methylation (*n* = 5), showed an increase of 4–19% in the mean CpG methylation in various CpG sites compared to controls, and three CpG sites were near the PAM motif of the sgRNA (Figure 8).

## Discussion

*In vivo* targeted epigenome modifications were reported in mouse (Lei et al., 2017), in the fish *Oryzias latipes* (Fukushima et al., 2019), and in the plant *Arabidopsis thaliana* (Ghoshal et al., 2020). dCas9 fused to the transcriptional activator domain VPR (dCas9-VPR) was used to activate transcription of a target gene *in vivo* in *Drosophila* (Lin et al., 2015) and targeted DNA methylation changes with a methylome engineering tool in invertebrates has been only reported in *Bombyx mori* (Liu et al., 2019).

The use of methylome engineering tools and *in vivo* transfection of our biological model, the snail *B. glabrata*, is technically challenging. One of the reasons is that the size of the embryo (50 µm diameter) is much smaller than in model species [e.g., zebrafish embryos 700 µm (Hanneman et al., 1988) and mice embryos 100 µm (Aigner et al., 2002)]. We developed a method of microinjection at the embryo yolk with an *in vivo* polymer-based reagent. Plasmids microinjected into the *B. glabrata* embryos produced transient expression of genes from the viral promoter SV40 for 5 days. These results indicate that plasmids were expressed without stable integration in the snail genome, SV40 promoter vectors can persist as nonreplicating extrachromosomal elements that are destroyed by nucleases, can be functionally inactivated by partitioning to non-nuclear compartments, or can be diluted by cell division (Cooper et al., 1997). Moreover, our study shows that dCas9-SunTag with DNMT3A-GFP transient expression (within 5 days’ timeframe of post-transfection) along with sgRNA complexity to dCas9 can perform site specific DNA methylation.

BSAS showed that the targeted methylation was achieved at low levels in some of the CpG sites of the transfected snails. In one transfected snail, five CpG sites were methylated between 3.5 and 4.4% while in another transfected snail also 4% of methylation was found but only in one CpG site. The other 3 transfected snails showed higher CpG methylation percentage principally in one CpG site (11–76%). Our findings indicate low DNA methylation in some CpG sites (∼4%) that is consistent with the idea that only some cells were transfected. It is now conceivable to increase the targeted DNA methylation by optimizing further the transfection at an earlier stage. Ten transfected embryos were compared to 9 control embryos (1 control bisulfite amplicon library yielded a very low number of reads) to confirm that the methylation change was only achieved in the transfected embryos. Control snails displayed less than 1% of CpG% in the 12 CpG sites of the targeted gene. Bisulfite conversion percentage was of ≥99% making it unlikely that we observed non-conversion artefacts at this precise target sequence.

The transfection technique presented in this work showed an efficiency of 4%, out of 250 microinjected embryos, 10 embryos showed the fluorescence of the reporter genes GFP and BFP and out of these 10 embryos, targeted methylation was achieved in 5 embryos, representing 2% of the total microinjected embryos, or 50% of the fluorescent positive embryos. The efficiency of the targeted DNA methylation could depend also on the dynamic folding of the chromatin, which regulates the accessibility to the DNA, and possibly some regions were accessible to the dCas9-SunTag-DNMT3 complex while others were not. As expected, mosaic DNA methylation patterns were found in the targeted gene, since the transfection was done at the gastrula stage. Two-cell transfection was attempted but the embryos did not survive the microinjection technique and the toxicity of the polymer-based reagent (Table 1). Such mosaicism of Cas9 action was also observed in *Crepidula fornicata*, the first mollusk species where genome editing has been achieved, even if the microinjection was done there at an earlier stage phase of the embryonic development (at the two-cell stage) (Perry and Henry, 2015). The high heterogeneity in targeted DNA methylation can be due to the dissimilar integration of vector constructions to the transfected cells, transfection is done at the embryo yolk and not at the embryo itself, and probably the integration of the vector to the embryo cells happens at different times. Additionally, we observed that the embryo development inside the eggs’ mass is not perfectly synchronized, which could also play a role in the differential vector integration to cells.

The dCas9-SunTag-DNMT3A1 was earlier transduced by lentiviral vectors to the HEK293T human cell line where it showed a high efficiency in inducing targeted DNA methylation, the increase was of 50–80% in 53 CpG sites of a CpG island along 600 bp of the gene *Hox5A*. The dCas9-SunTag-DNMT3A1 system was compared to the vector dCas9-DNMT3A, the first one showed an increase of 60–95% of the CpG methylation in a 4-kb window while the second one showed an increase of 20–40% at the same 4-kb window. These results showed that the dCas9-SunTag-DNMT3A1 system showed higher efficiency in inducing a targeted DNA methylation than dCas9-DNMT3A (Huang et al., 2017).

The targeted modification *in vivo* of DNA methylation has been mostly addressed in the mice model. For example, the use of dCas9 fused to the prokaryotic methyltransferase MQ1 (dCas-MQ1) was tested *in vivo* in mouse zygotes where it induced an increase from 50% CpG methylation to 70% in 20 CpG sites along 500 bp of the locus *Igf2/H19* (Lei et al., 2017).

Comparisons of the results found *in vitro* in a human cell line, showed that, in our case, the targeted DNA methylation increase was weaker and highly heterogeneous. A major difference is that our transfections were performed *in vivo* at the gastrula stage and not at the one cell stage or in a cell culture. *In vivo* transfection efficiencies are not as high as viral vectors used *in vitro*, nucleic acids are more stable in cell culture and integrate more efficiently in one cell nucleus, but in a living organism, they can be degraded before cell integration.

It is more suitable to compare our increased targeted DNA methylation results to that obtained *in vivo* in mouse zygotes with the vector dCas9-MQ1 (Lei et al., 2017). Indeed, our results are similar: in mice the global increase was of 10–60% in 7 CpG sites using a unique sgRNA while in our model the increase was of ∼4–76% in up to 5 CpG sites. In the mouse zygotes, the range of the CpG methylation increase was improved by using a pool of 4 sgRNAs, increasing the CpG% to 20–50% in 12 CpG sites. In the case of dCas9-DNMT3 vector, co-transfection of multiple sgRNAs also revealed an increase in the range of the targeted CpG% modification compared to the use of a unique sgRNA (Lei et al., 2017); one sgRNA increases the methylation 30–60% in 6 CpG sites, and when 4 sgRNAs were co-transfected the methylation increased 25–55% in 12 CpG sites. Therefore, to increase the range of the DNA methylation effect, multiple sgRNAs targeting the same *locus* should be used in the future. We estimate that at least 4 efficient sgRNAs must be developed for a target region of ∼500 bp.

Another invertebrate in which targeted DNA methylation has been modified is the silkworm *Bombyx mori*, where dCas9-TET1 was used to demethylate the gene body *in vitro* and *in vivo* (Liu et al., 2019). The *in vitro* demethylation percentages were 30–60% in 9 of 9 CpG sites of the gene *BGIBMGA004109* and 10–42% in 8 of 9 CpG sites of the gene *BGIBMGA001471*. The *in vivo* demethylation was achieved in 5 of 9 CpG sites of the gene *BGIBMGA004109*, the 5 CpG sites displayed different DNA demethylation percentages from 20 to 50%. In the gene *BGIBMGA001471* the CpG demethylation *in vivo* was achieved in 7 of 9 CpG sites, with a methylation decrease from 5 to 40%. The editing range was within 200 bp, and the editing efficiency was lower in embryo than in embryonic cell line. Furthermore, gene body demethylation was found to promote transcription in the *B. mori* embryonic cell line which is contradictory to the hypothesis that GBM is correlated with higher gene expression; nevertheless, further work is needed to explore the causal relationship between GBM and transcription, particularly in invertebrate taxa.

Our work showed that the use of epigenetic engineering tools *in vivo* in the snail *B. glabrata* and probably other mollusks species is now conceivable. The further development of transfection and epigenetic engineering tools in our biological model will bring insights about the role of GBM in invertebrate species, a subject which requires experimental tools to address this question. While not formally tested, our technique could probably also be used for Cas9-based gene editing.

## Data Availability

The datasets presented in this study can be found in online repositories. The names of the repository/repositories and accession number(s) can be found below: https://www.ncbi.nlm.nih.gov/, PRJNA770996. https://doi.org/10.5281/zenodo.5786326, 10.5281/zenodo.5786326. https://doi.org/10.5281/zenodo.5786314, 10.5281/zenodo.5786314. https://doi.org/10.5281/zenodo.4277533, 10.5281/zenodo.4277533.

## References

[B1] Aigner A. Ray P. E. Czubayko F. Wellstein A. (2002). Immunolocalization of an FGF-Binding Protein Reveals a Widespread Expression Pattern during Different Stages of Mouse Embryo Development. Histochem. Cel Biol 117 (1), 1–11. 10.1007/s00418-001-0360-4 11819092

[B2] Augusto R. D. C. Duval D. Grunau C. (2019). Effects of the Environment on Developmental Plasticity and Infection Success of *Schistosoma* Parasites - an Epigenetic Perspective. Front. Microbiol. 10, 1475. 10.3389/fmicb.2019.01475 31354641 PMC6632547

[B3] Brezgin S. Kostyusheva A. Kostyushev D. Chulanov V. (2019). Dead Cas Systems: Types, Principles, and Applications. Ijms 20 (23), 6041. 10.3390/ijms20236041 PMC6929090 31801211

[B4] Camey T. Verdonk N. H. (1969). The Early Development of the Snail *Biomphalaria glabrata* (Say) and the Origin of the Head Organs. Neth. J. Zool 20 (1), 93–121. 10.1163/002829670x00097

[B5] Chiappinelli K. B. Strissel P. L. Desrichard A. Li H. Henke C. Akman B. Hein A. Rote N. S. Cope L. M. Snyder A. Makarov V. Buhu S. Slamon D. J. Wolchok J. D. Pardoll D. M. Beckmann M. W. Zahnow C. A. Merghoub T. Chan T. A. Baylin S. B. Strick R. (2015). Inhibiting DNA Methylation Causes an Interferon Response in Cancer via dsRNA Including Endogenous Retroviruses. Cell 162 (5), 974–986. 10.1016/j.cell.2015.07.011 26317466 PMC4556003

[B6] Clement K. Rees H. Canver M. C. Gehrke J. M. Farouni R. Hsu J. Y. (2019). CRISPResso2 Provides Accurate and Rapid Genome Editing Sequence Analysis. Nat. Biotechnol. 37 (3), 224–226. 10.1038/s41587-019-0032-3 30809026 PMC6533916

[B7] Coelho F. S. Rodpai R. Miller A. Karinshak S. E. Mann V. H. dos Santos Carvalho O. (2020). Diminished Adherence of *Biomphalaria glabrata* Embryonic Cell Line to Sporocysts of *Schistosoma Mansoni* Following Programmed Knockout of the Allograft Inflammatory Factor. Parasites Vectors 13 (1), 1–12. 10.1186/s13071-020-04384-9 33050923 PMC7552541

[B8] Colley D. G. Bustinduy A. L. Secor W. E. King C. H. (2014). Human Schistosomiasis. The Lancet 383 (9936), 2253–2264. 10.1016/S0140-6736(13)61949-2 PMC4672382 24698483

[B9] Cooper M. J. Lippa M. Payne J. M. Hatzivassiliou G. Reifenberg E. Fayazi B. (1997). Safety-modified Episomal Vectors for Human Gene Therapy. Proc. Natl. Acad. Sci. 94 (12), 6450–6455. 10.1073/pnas.94.12.6450 9177238 PMC21070

[B10] Duval D. Galinier R. Portela J. Mitta G. Gourbal B. (2013). Immunocytochemical Detection of Recombinant Biomphalysin on *Schistosoma Mansoni* Sporocysts. Bio-protocol 3 (22), e969. 10.21769/BioProtoc.969

[B11] Esteller M. (2002). CpG Island Hypermethylation and Tumor Suppressor Genes: a Booming Present, a Brighter Future. Oncogene 21 (35), 5427–5440. 10.1038/sj.onc.1205600 12154405

[B12] Fukushima H. S. Takeda H. Nakamura R. (2019). Targeted *In Vivo* Epigenome Editing of H3K27me3. Epigenetics & Chromatin 12 (1), 1–12. 10.1186/s13072-019-0263-z 30871638 PMC6419334

[B13] Galinier R. Portela J. Moné Y. Allienne J. F. Henri H. Delbecq S. (2013). Biomphalysin, a New β Pore-Forming Toxin Involved in *Biomphalaria glabrata* Immune Defense against *Schistosoma Mansoni* . Plos Pathog. 9 (3), e1003216. 10.1371/journal.ppat.1003216 23555242 PMC3605176

[B14] Galinier R. Roger E. Moné Y. Duval D. Portet A. Pinaud S. (2017). A Multistrain Approach to Studying the Mechanisms Underlying Compatibility in the Interaction between *Biomphalaria glabrata* and *Schistosoma Mansoni* . Plos Negl. Trop. Dis. 11 (3), e0005398. 10.1371/journal.pntd.0005398 28253264 PMC5349689

[B15] Geyer K. K. Niazi U. H. Duval D. Cosseau C. Tomlinson C. Chalmers I. W. (2017). The *Biomphalaria glabrata* DNA Methylation Machinery Displays Spatial Tissue Expression, Is Differentially Active in Distinct Snail Populations and Is Modulated by Interactions with *Schistosoma Mansoni* . Plos Negl. Trop. Dis. 11 (5), e0005246. 10.1371/journal.pntd.0005246 28510608 PMC5433704

[B16] Ghoshal B. Vong B. Picard C. L. Feng S. Tam J. M. Jacobsen S. E. (2020). A Viral Guide RNA Delivery System for CRISPR-Based Transcriptional Activation and Heritable Targeted DNA Demethylation in *Arabidopsis thaliana* . Plos Genet. 16 (12), e1008983. 10.1371/journal.pgen.1008983 33315895 PMC7769603

[B17] Gómez-Díaz E. Jordà M. Peinado M. A. Rivero A. (2012). Epigenetics of Host-Pathogen Interactions: The Road Ahead and the Road behind. Plos Pathog. 8 (11), e1003007. 10.1371/journal.ppat.1003007 23209403 PMC3510240

[B18] Gryseels B. Polman K. Clerinx J. Kestens L. (2006). Human Schistosomiasis. The Lancet 368 (9541), 1106–1118. 10.1016/S0140-6736(06)69440-3 16997665

[B19] Hanford H. E. Von Dwingelo J. Abu Kwaik Y. (2021). Bacterial Nucleomodulins: A Coevolutionary Adaptation to the Eukaryotic Command center. Plos Pathog. 17 (1), e1009184. 10.1371/journal.ppat.1009184 33476322 PMC7819608

[B20] Hanneman E. Trevarrow B. Metcalfe W. K. Kimmel C. B. Westerfield M. (1988). Segmental Pattern of Development of the Hindbrain and Spinal Cord of the Zebrafish Embryo. Development 103 (1), 49–58. 10.1242/dev.103.1.49 3197633

[B21] Herricks J. R. Hotez P. J. Wanga V. Coffeng L. E. Haagsma J. A. Basáñez M.-G. (2017). The Global burden of Disease Study 2013: What Does it Mean for the NTDs? Plos Negl. Trop. Dis. 11 (8), e0005424. 10.1371/journal.pntd.0005424 28771480 PMC5542388

[B22] Hinman V. F. O'Brien E. K. Richards G. S. Degnan B. M. (2003). Expression of Anterior Hox Genes during Larval Development of the Gastropod Haliotis Asinina. Evol. Dev. 5 (5), 508–521. 10.1046/j.1525-142X.2003.03056.x 12950629

[B23] Holtzman L. Gersbach C. A. (2018). Editing the Epigenome: Reshaping the Genomic Landscape. Annu. Rev. Genom. Hum. Genet. 19, 43–71. 10.1146/annurev-genom-083117-021632 29852072

[B24] Huang Y.-H. Su J. Lei Y. Brunetti L. Gundry M. C. Zhang X. (2017). DNA Epigenome Editing Using CRISPR-Cas SunTag-Directed DNMT3A. Genome Biol. 18 (1), 1–11. 10.1186/s13059-017-1306-z 28923089 PMC5604343

[B25] Iijima M. Akiba N. Sarashina I. Kuratani S. Endo K. (2006). Evolution of Hox Genes in Molluscs: a Comparison Among Seven Morphologically Diverse Classes. J. molluscan Stud. 72 (3), 259–266. 10.1093/mollus/eyl001

[B26] Jiang M. Zhang Y. Fei J. Chang X. Fan W. Qian X. (2010). Rapid Quantification of DNA Methylation by Measuring Relative Peak Heights in Direct Bisulfite-PCR Sequencing Traces. Lab. Invest. 90 (2), 282–290. 10.1038/labinvest.2009.132 20010852

[B27] Jing L. (2012). Zebrafish Embryo DNA Preparation. Bio-protocol 2, e184. 10.21769/BioProtoc.184

[B28] Jones P. A. (2012). Functions of DNA Methylation: Islands, Start Sites, Gene Bodies and beyond. Nat. Rev. Genet. 13 (7), 484–492. 10.1038/nrg3230 22641018

[B29] Keller T. E. Han P. Yi S. V. (2016). Evolutionary Transition of Promoter and Gene Body DNA Methylation across Invertebrate-Vertebrate Boundary. Mol. Biol. Evol. 33 (4), 1019–1028. 10.1093/molbev/msv345 26715626 PMC4776710

[B30] Kenny N. J. Truchado-García M. Grande C. (2016). Deep, Multi-Stage Transcriptome of the Schistosomiasis Vector *Biomphalaria glabrata* Provides Platform for Understanding Molluscan Disease-Related Pathways. BMC Infect. Dis. 16 (1), 1–10. 10.1186/s12879-016-1944-x 27793108 PMC5084317

[B31] King C. H. Dangerfield-Cha M. (2008). The Unacknowledged Impact of Chronic Schistosomiasis. Chronic illness 4 (1), 65–79. 10.1177/1742395307084407 18322031

[B32] Knight M. Ittiprasert W. Arican-Goktas H. D. Bridger J. M. (2016). Epigenetic Modulation, Stress and Plasticity in Susceptibility of the Snail Host, *Biomphalaria glabrata*, to *Schistosoma Mansoni* Infection. Int. J. Parasitol. 46 (7), 389–394. 10.1016/j.ijpara.2016.03.003 27056272

[B33] Lei Y. Huang Y.-H. Goodell M. A. (2018). DNA Methylation and De-methylation Using Hybrid Site-Targeting Proteins. Genome Biol. 19 (1), 1–12. 10.1186/s13059-018-1566-2 30400938 PMC6219187

[B34] Lei Y. Zhang X. Su J. Jeong M. Gundry M. C. Huang Y.-H. (2017). Targeted DNA Methylation *In Vivo* Using an Engineered dCas9-MQ1 Fusion Protein. Nat. Commun. 8 (1), 1–10. 10.1038/ncomms16026 28695892 PMC5508226

[B35] Lin S. Ewen-Campen B. Ni X. Housden B. E. Perrimon N. (2015). *In Vivo* transcriptional Activation Using CRISPR/Cas9 in Drosophila. Genetics 201 (2), 433–442. 10.1534/genetics.115.181065 26245833 PMC4596659

[B36] Liu Y. Ma S. Chang J. Zhang T. Chen X. Liang Y. (2019). Programmable Targeted Epigenetic Editing Using CRISPR System in *Bombyx mori* . Insect Biochem. Mol. Biol. 110, 105–111. 10.1016/j.ibmb.2019.04.013 31022512

[B37] Luviano N. Lopez M. Gawehns F. Chaparro C. Arimondo P. Ivanovic S. (2021). The Methylome of *Biomphalaria glabrata* and Other Mollusks: Enduring Modification of Epigenetic Landscape and Phenotypic Traits by a New DNA Methylation Inhibitor. Epigenetics and Chromatin 14, 48. 10.1186/s13072-021-00422-7 34702322 PMC8549274

[B38] Mehravar M. Shirazi A. Mehrazar M. M. Nazari M. (2019). *In Vitro* pre-validation of Gene Editing by CRISPR/Cas9 Ribonucleoprotein. Avicenna J. Med. Biotechnol. 11 (3), 259–263. 31380000 PMC6626505

[B39] Melillo D. Marino R. Italiani P. Boraschi D. (2018). Innate Immune Memory in Invertebrate Metazoans: a Critical Appraisal. Front. Immunol. 9, 1915. 10.3389/fimmu.2018.01915 30186286 PMC6113390

[B40] Montague T. G. Cruz J. M. Gagnon J. A. Church G. M. Valen E. (2014). CHOPCHOP: a CRISPR/Cas9 and TALEN Web Tool for Genome Editing. Nucleic Acids Res. 42 (W1), W401–W407. 10.1093/nar/gku410 24861617 PMC4086086

[B41] Netea M. G. Joosten L. A. B. Latz E. Mills K. H. G. Natoli G. Stunnenberg H. G. (2016). Trained Immunity: a Program of Innate Immune Memory in Health and Disease. Science 352 (6284), aaf1098. 10.1126/science.aaf1098 27102489 PMC5087274

[B42] Oka M. Rodić N. Graddy J. Chang L.-J. Terada N. (2006). CpG Sites Preferentially Methylated by Dnmt3a *In Vivo* . J. Biol. Chem. 281 (15), 9901–9908. 10.1074/jbc.M511100200 16439359

[B43] Pearce E. J. MacDonald A. S. (2002). The Immunobiology of Schistosomiasis. Nat. Rev. Immunol. 2 (7), 499–511. 10.1038/nri843 12094224

[B44] Perry K. J. Henry J. Q. (2015). CRISPR/Cas9-mediated Genome Modification in the mollusc,Crepidula Fornicata. genesis 53 (2), 237–244. 10.1002/dvg.22843 25529990

[B45] Plummer R. J. Guo Y. Peng Y. (2018). A CRISPR Reimagining: New Twists and Turns of CRISPR beyond the Genome‐engineering Revolution. J. Cel. Biochem. 119 (2), 1299–1308. 10.1002/jcb.26406 28926145

[B46] Roger E. Gourbal B. Grunau C. Pierce R. J. Galinier R. Mitta G. (2008). Expression Analysis of Highly Polymorphic Mucin Proteins (Sm PoMuc) from the Parasite *Schistosoma Mansoni* . Mol. Biochem. Parasitol. 157 (2), 217–227. 10.1016/j.molbiopara.2007.11.015 18187213

[B47] Roquis D. Taudt A. Geyer K. K. Padalino G. Hoffmann K. F. Holroyd N. (2018). Histone Methylation Changes Are Required for Life Cycle Progression in the Human Parasite *Schistosoma Mansoni* . Plos Pathog. 14 (5), e1007066. 10.1371/journal.ppat.1007066 29782530 PMC5983875

[B48] Sarda S. Zeng J. Hunt B. G. Yi S. V. (2012). The Evolution of Invertebrate Gene Body Methylation. Mol. Biol. Evol. 29 (8), 1907–1916. 10.1093/molbev/mss062 22328716

[B55] Shan S. Soltis P. Soltis D. E. Yang B. (2020). Considerations in adapting CRISPR/Case9 in nongenetic Model Plant Systems. Applications in Plant Sciences 8 (1), e11314. 31993256 10.1002/aps3.11314 PMC6976890

[B49] Tanenbaum M. E. Gilbert L. A. Qi L. S. Weissman J. S. Vale R. D. (2014). A Protein-Tagging System for Signal Amplification in Gene Expression and Fluorescence Imaging. Cell 159 (3), 635–646. 10.1016/j.cell.2014.09.039 25307933 PMC4252608

[B50] Theron A. Rognon A. Gourbal B. Mitta G. (2014). Multi-parasite Host Susceptibility and Multi-Host Parasite Infectivity: a New Approach of the *Biomphalaria glabrata/Schistosoma Mansoni* Compatibility Polymorphism. Infect. Genet. Evol. 26, 80–88. 10.1016/j.meegid.2014.04.025 24837670

[B51] Vojta A. Dobrinić P. Tadić V. Bočkor L. Korać P. Julg B. (2016). Repurposing the CRISPR-Cas9 System for Targeted DNA Methylation. Nucleic Acids Res. 44 (12), 5615–5628. 10.1093/nar/gkw159 26969735 PMC4937303

[B56] World Health Organization (WHO) (2020). World Health Statistics..

[B52] Wu X. Kriz A. J. Sharp P. A. (2014). Target Specificity of the CRISPR-Cas9 System. Quant Biol. 2 (2), 59–70. 10.1007/s40484-014-0030-x 25722925 PMC4338555

[B53] Zaboikin M. Zaboikina T. Freter C. Srinivasakumar N. (2017). Non-homologous End Joining and Homology Directed DNA Repair Frequency of Double-Stranded Breaks Introduced by Genome Editing Reagents. PloS one 12 (1), e0169931. 10.1371/journal.pone.0169931 28095454 PMC5241150

[B54] Zemach A. McDaniel I. E. Silva P. Zilberman D. (2010). Genome-wide Evolutionary Analysis of Eukaryotic DNA Methylation. Science 328 (5980), 916–919. 10.1126/science.1186366 20395474

